# Neighborhood Walkability and Physical and Mental Health among Older Adults Living in a Deprived Community

**DOI:** 10.1093/geroni/igab046.2361

**Published:** 2021-12-17

**Authors:** Jennifer Blackwood, Rie Suzuki, Shailee Shah, Noah Webster

**Affiliations:** 1 University of Michigan--Flint, Michigan, United States; 2 University of Michigan-Flint, Flint, Michigan, United States; 3 University of Michigan-Flint, University of Michigan-Flint, Michigan, United States; 4 University of Michigan, Ann Arbor, Michigan, United States

## Abstract

Background: Identifying the factors to improve the quality of life (QOL) is vital to decrease morbidity and mortality rates among older adults. Although unfavorable neighborhood features have a significant negative impact on QOL, few studies have investigated these relationships in a deprived community. The purpose of the study was to understand how neighborhood walkability is associated with QOL using the SF-36 among urban-dwelling older adults. Methods: This is a cross-sectional survey. Participants were recruited in 2018 and 2019 at regional health clinics in Flint, MI. To be eligible, participants had to be over 65 years old and Flint residents. Results: Of the 132 participants, the majority were female (66%), African American (77%), single, divorced, or widowed (75%), and educated below GED level (84%). After adjusting for gender, assistive device use, medication, and the Supplemental Security Income receipt, multiple regression analysis revealed that those with better perceptions of land-mixed use and accessibility within their neighborhood were more likely to have better physical health (β = .36, p<.05). However, the perceptions of greater pedestrian safety were associated with the poor physical and mental health (PCS; β = -0.19, p <.05; MCS; β = -0.25, p < .05). Perceptions of the presence of walking hazards and crime were not significantly associated with QOL. Discussion: Findings suggest that neighborhood walkability characteristics are associated with physical health. The development of walking programs with accessible neighborhoods will be urgent to improve the health-related QOL for older adults living in a targeted community.

